# The development and face validity of the music therapy sensory instrument for cognition, consciousness, and awareness (MuSICCA)

**DOI:** 10.3389/fpsyg.2025.1441178

**Published:** 2025-05-06

**Authors:** Jonathan W. Pool, Wendy L. Magee, Richard J. Siegert, Claire L. Wood

**Affiliations:** ^1^Cambridge Institute for Music Therapy Research, Anglia Ruskin University, Cambridge, United Kingdom; ^2^Boyer College of Music and Dance, Temple University, Philadelphia, PA, United States; ^3^Department of Psychology and Neuroscience, School of Clinical Sciences, Faculty of Health and Environmental Science, Auckland University of Technology, Auckland, New Zealand

**Keywords:** MuSICCA, disorders of consciousness, music, assessment, face validity, pediatric, children, adolescents

## Abstract

Severe brain injuries in children and young people can result in disorders of consciousness. This can pose significant challenges for the brain injury survivor as they may struggle to show awareness; for their family, who want to help their child to recover consciousness; and for the team providing treatment and care for them, who need an assessment that will inform optimal treatment and care planning. Currently, there is a paucity of fully validated behavioral tools to assess consciousness in 2–18-year-olds. Assessing awareness across this age range is challenging and complex due to neurodevelopmental changes that occur during maturation. This study evaluated the face validity of a music-based behavioral assessment for children and young people with disorders of consciousness. This is known as the Music therapy Sensory Instrument for Cognition, Consciousness and Awareness (MuSICCA). The study recruited 20 participants to compose a mixed cohort of music therapists, non-music therapy healthcare professionals and family members with lived experience of caring for a child or young person with a disorder of consciousness. These participants reviewed the MuSICCA and evaluated its suitability as an assessment of consciousness for use with children and young people. They provided feedback by rating their level of agreement with two statements and they also described the perceived strengths and limitations of the MuSICCA. The results showed substantial agreement among raters that the MuSICCA appears to be an assessment of consciousness and awareness, and that the MuSICCA appears to be suitable for use with children and young people. Its strengths include being rigorous, comprehensive, providing guidance and opportunity for caregiver involvement, its use of salience in stimulation, and its utility in supporting the wider clinical and care teams. The findings suggest that the MuSICCA may be a valuable assessment tool in providing treatment and care for children and young people with disorders of consciousness and their families.

## 1 Introduction

Acquired Brain Injury (ABI) may be fatal, severe, moderate, or mild. Severe ABI can result in Disorders of Consciousness (DoC) which are characterized by difficulties/problems with levels of wakefulness and awareness. The disorders within this category include Vegetative State (VS), also known as Unresponsive Wakefulness Syndrome (UWS), and Minimally Conscious State (MCS; Royal College of Physicians, 2020). The behavioral characteristics of VS/UWS comprise of spontaneous arousal and sleep-wake cycles; no sustained, reproducible purposeful or voluntary behavioral responses to sensory stimuli; and an absence of demonstrated awareness of self or others in their environment (Multi-Society Task Force on PVS, 1994; Royal College of Physicians, 2020). The behavioral repertoire of VS/UWS suggests neurological activity that is reflexive and mainly at the sub-cortical level. The minimally consciousness state is characterized by diminished consciousness, but where the patient's behaviors demonstrate some awareness of themself, in addition to behaviors that are reproducible and linked to specific environmental stimuli suggesting awareness of others (Royal College of Physicians, 2020). These behaviors suggest activity in the brain recruiting the cortex, and therefore, implying the presence of cognitively mediated responses.

Advances in medicine are leading to increasing numbers of people surviving severe brain injuries, leaving some with substantially impaired consciousness. The population of people with DoC have complex needs and multiple disabilities. Measuring responsiveness is a topic of considerable interest, as the information generated from the range of validated behavioral measures (Seel et al., 2010) is used to inform care and treatment planning by the whole multidisciplinary team. Inaccurate assessment continues to be a significant issue for the multidisciplinary teams working with people with DoC (Wang et al., 2020). The consequences of misdiagnosis include inadequate provision of care, treatment programmes that are not optimized for maximizing functional gains in areas of potential, poor identification of the patient's intentions to communicate, and insufficient evidence to support challenging decision-making around life support (withdrawal of hydration and nutrition) and resuscitation (Ashwal, 2013; Ashwal and Cranford, 2002). Therefore, it is fundamental to develop and validate assessment tools to accurately assess this population, thus improving the care, treatment, and prognosis for people with DoC.

The process of assessing awareness in DoC is complex and made more challenging when assessing children and young people with these disorders. This added complexity is due to developmental factors, involving changes in language, cognition, and motor ability, which vary across age groups and within them. This can be a major challenge for clinicians and the family who need accurate assessment information regarding awareness and function to determine realistic expectations of the patient's abilities (Menén Sánchez et al., 2023; Alvarez et al., 2019). Best practice guidelines advise that pediatric neurorehabilitation should be family-centered, and that family involvement can enhance the outcome of assessments (Centers for Disease Control Prevention, 2018). Treatments for children and young people should also draw on identity-based rehabilitation frameworks (Perkins et al., 2022). Music-based assessment protocols provide a plausible alternative to language-based ones, as they do not rely on language processing ability. Furthermore, music is culturally ubiquitous and often used in pedagogical environments, leisure, and religious contexts (Pool and Magee, 2016). In adults, music is known to increase motivation (Dimitriadis et al., 2023), and influence awareness-related outcomes in DoC patients, including promoting arousal and attention (O'Kelly et al., 2013), boosting cognition (Castro et al., 2015) and improving measures of awareness (Verger et al., 2014).

At the time of writing, there is a paucity of fully validated awareness assessment tools for children and young people with DoC, although some measures have “preliminary validation” (Molteni et al., 2023). One assessment of awareness for adults is validated: the Music therapy Assessment Tool for Awareness in Disorders Of Consciousness (MATADOC). Its principal subscale has demonstrated good inter-rater reliability (mean = 0.83, SD = 0.11), good test–retest reliability (mean = 0.82, SD = 0.05), and good internal consistency (α = 0.76; Magee et al., 2014). The MATADOC has fair to moderate concurrent validity with the criterion standard Coma Recovery Scale-Revised but measures awareness in different yet complementary ways due to its reliance on non-verbal music stimuli (Magee et al., 2023). The MATADOC protocol utilizes the presentation of a range of music stimuli, including those that are emotionally salient such as singing the patient's name as well as personally meaningful songs (Amari et al., 2017; Menén Sánchez et al., 2023). This supports its relevance for use with children also. The MATADOC was shown to provide a clinically useful protocol and measure for behavioral assessment and clinical treatment planning with children with DoC (Magee et al., 2015). However, it required some modifications and validation for use with this population.

Therefore, a pediatric version of the MATADOC was developed called the Music therapy Sensory Instrument for Cognition, Consciousness and Awareness (MuSICCA). It was developed clinically in consultation with a stakeholder group. This consisted of parents of children with DoC and professionals working in pediatric DoC settings. This multiprofessional group included educational psychologists, clinical psychologists, speech and language therapists, occupational therapists, physiotherapists, educators, and a global network of music therapists experienced in pediatric DoC who were MATADOC-trained. The MuSICCA protocol expands on that of the MATADOC and involves a minimum of six tasks using musical stimulation and non-stimulated observation periods of 3 min each before and after the stimulation period. Furthermore, the MuSICCA protocol offers family members the opportunity to be actively involved in delivering the procedures, thus maximizing salience within the stimuli presented and drawing on intimate knowledge of the child. The child is provided with opportunities to listen to music that is played live and based on the child's behaviors (e.g., breathing rate; motor behaviors), to show physical responses, vocalize, and make sounds or choices of music to be played by the therapist. Responses are rated across the motor, visual, auditory, communication, and arousal domains in 15 items. The MuSICCA is designed to provide an indicative diagnosis for children with DoC alongside other measures. It provides detailed information about their responses to stimuli to support the multidisciplinary treatment team and will contribute to the effort to reduce instances of misdiagnosis.

When testing a new measure to determine its validity and reliability, face validity is an important first step in the process. However, despite its importance, many studies fail to report on face validity testing in detail.

Validity might be broadly defined as all the quantitative and qualitative evidence we can muster to attest that a psychometric measure actually measures what it purports to measure. Streiner and Norman (2008) note that traditionally many textbooks have taught this topic in terms of the ‘three Cs' namely *content, criterion*, and *construct validity*. Content validity is the extent to which a tool or measure taps into all the important elements of the construct of interest—such as whether a class test in mathematics samples all the major topics covered by the teacher in class. Criterion validity is the extent to which the measure correlates positively with other tests of a similar construct (convergent) or negatively with tests of a dissimilar (divergent) construct. Criterion validity can also refer to the ability of the measure to predict future outcomes. For example, we might test whether a measure of early infant temperament can predict subsequent development of emotional problems in adolescence. The third C, construct validity can be thought of as the extent to which all the data on a specific measure suggests that scores on that measure conform to or support our theoretical model of that construct. Some authors have gone so far as to argue that all forms of validity then constitute evidence for construct validity (Borsboom, 2005). However, a fourth important class of validity, and the focus of the present study is that of *face* validity.

Face validity is typically described as the extent to which a psychometric instrument appears acceptable, credible, sensible, reasonable, and plausible to the person responding to it. In a seminal paper on the topic (Mosier, 1947) proposed four major types of face validity; (i) validity by assumption, (ii) by definition, (iii) by hypothesis, and (iv) the appearance of validity. In an editorial on the issue of face validity three quarters of a century later (Allen et al., 2023) observe that contemporary definitions of face validity are almost entirely concerned with the latter—that the items in any measure appear valid to the test-takers. They identify Holden's (2010) definition of face validity as the most up to date: “Face validity refers to a characteristic associated with a psychological test and its individual items. Distinct from more technical types of validity, face validity is the appropriateness, sensibility, or relevance of the test and its items as they appear to the person answering the test. That is, do a test and its items look valid and meaningful to the individual taking the test? More formally, face validity has been defined as the degree to which a test respondent views the content of a test and its items as relevant to the situation being considered (Holden, 2010, p. 637).”

In this respect it could be argued that the present study is *not* about face validity—at least not in the sense as defined by Holden. We are concerned here with a group of patients, children with disorders of consciousness who are by and large not able to respond to simple requests or instructions and incapable of giving their impressions and reactions to the items of the MuSICCA. Consequently, we made the decision to assess the face validity of the MuSICCA and its items by proxy using music therapists and other professionals experienced in working with children with disorders of consciousness, and the parents of such children. Allen et al. (2023) suggest that face validity has often been treated as less important than those aspects of validity that lend themselves more readily to quantitative assessment. They also argue that it is an essential component of scale development and one which has particular importance for measures intended for use with children, clinical conditions, and disabled persons. In the present study, we worked with family caregivers, non-music therapy professionals working in pediatric DoC, and experienced pediatric music therapists to examine how they perceived the face validity of the MuSICCA.

This present study of face validity described here forms the first phase of a much larger and more detailed evaluation of the MuSICCA. The larger study will evaluate the MuSICCA's construct validity, its inter-rater and test-retest reliability, and its clinical utility. For details about the overall research project, please refer to the published protocol (Pool et al., 2020) that has also received ethical approval from the Research Ethics Committee and Health Research Authority of the National Health Service of the UK (ID: 167534).

The research questions for this study of face validity are:

Does the MuSICCA have sufficient face validity to be an assessment of consciousness and awareness and suitable for use with children and young people?
What are its strengths and limitations from the perspectives of trained music therapists, other healthcare professionals, and family caregivers?

## 2 Method

### 2.1 Recruitment and description of participants

Twenty participants were recruited to evaluate the face validity of the MuSICCA. The selection of participants was carried out according to the following criteria:

#### 2.1.1 Inclusion criteria

- The participant needed to have experience of working with or caring for children and young people with a DoC.
- The participants were one of the following:

° a registered music therapist trained in the MATADOC or
° a person with lived experience as a family caregiver of a child and young people with a DoC or
° a non-music therapy healthcare professional.

#### 2.1.2 Exclusion criteria

All members of the research team were excluded from being participants.

The music therapists were recruited from the database of MATADOC-trained professionals with experience of working with children with acquired brain injuries. The families and other healthcare professionals were recruited from a specialist pediatric rehabilitation center in the UK. These participants were invited to review the MuSICCA video demonstration. This consisted of a 30-min verbal description accompanied by video excerpts of the procedure, followed by 30 min for a question and answer session. The video of the material is available by request from the corresponding author. The respondents were asked to provide feedback about the MuSICCA's face validity in response to two statements:

Statement 1: On initial review, the MuSICCA appears to be an assessment of consciousness and awareness.
Statement 2: On initial review, the MuSICCA appears to be suitable for use with children and young people.

Respondents independently rated their level of agreement or disagreement with each statement by selecting one of the following: strongly disagree, disagree, undecided, agree, and strongly agree. In addition to this, the respondents were asked for free text comments about the perceived strengths and limitations of the MuSICCA. Answers were submitted electronically via email, or in hard copy form depending on convenience. All anonymised response forms were printed as hard copies and stored securely in a locked cabinet.

The level of agreement between respondents regarding the statements was determined as a quantitative measure of face validity for the MuSICCA. In this study, the minimum level of agreement between reviewers was set at 75% for the MuSICCA to be considered to have face validity (Pool et al., 2020). This criterion for each statement was met if 75% of respondents agreed or strongly agreed. If this was achieved for both statements, then the MuSICCA would be considered ready for further testing for validity, inter-rater and test-retest reliability, and clinical utility. In this way, this study formed part of a more comprehensive process of developing and validating this new measure.

### 2.2 Data analysis

The quantitative data from the respondents' ratings to the statements were analyzed using percentage level of agreement calculated in Microsoft Excel, and Gwet's (2008) AC1 for measuring level of agreement between raters. This measure of agreement was selected *post-hoc* for its suitability for five-point agreement scale involving multiple raters and few items with the raw data indicating high agreement. This was calculated using R statistical software. The qualitative free text responses regarding the strengths and limitations of the MuSICCA were analyzed using thematic analysis (Braun and Clarke, 2012). Coding was carried out by hand without the use of software. The purpose of thematic analysis in this study was to enable categorization of the strengths and limitations and detect relationships between themes. In this process, the free text comments from the questionnaires were presented in a table. Then, they were analyzed independently by two of the research team (JWP & WLM) using a theoretical (deductive) approach driven by their knowledge of the MuSICCA, the published literature in this area and the population of people with disorders of consciousness. Coding occurred at the latent level and was interpretive to allow analysis to examine the underlying theoretical ideas that shaped and informed the semantic content. Thus, initial codes (category themes) were created independently (JWP & WLM) and recorded in separate tables containing the original free text comments and initial codes. Then, the analysts met to discuss these initial codes until consensus was reached on a refined set of codes and themes. These were then reviewed by one of the original respondents (CW) to determine their fidelity, usefulness, relevance, and trustworthiness. The two analysts and the respondent discussed the latest codes and themes, refining these further to produce a final set of codes and themes. In this process, a simple thematic map of codes and themes was used to conceptualize the data patterns and the relationships between them. Codes and themes were merged to form broader, more comprehensive ones, while others maintained a discrete, narrower meaning. Table 1 shows a sample from the codebook. Then, the free text extracts were checked to consider whether these formed a pattern within each theme that was logical and consistent. The validity of the themes was considered in relation to the full data set and the thematic map was used to test these relationships. This process concluded with the definition and distillation of the themes and the organized thematic collation of data extracts for reporting on the findings. These were then related to the experience of the respondent (CW) who is a music therapist with experience of assessing and providing therapy for children and young people with disorders of consciousness and has trialed the MuSICCA for this study.

**Table 1:** Codebook excerpt from thematic analysis.

**Raw data**	**Description**	**Initial theme**	**Superordinate theme**	**Domain**
“Children should have parents present during the assessment”	Need for parental involvement during assessments	Involving caregivers	Benefits and risks of caregiver involvement	Utility
“A validated tool that will allow assessment and then go on to inform a treatment plan (this will/should offer reassurance to parents that assessment is meaningful and not an end state)”	An assessment tool that will inform goal-setting and seem useful to parents	Informs treatment and goal-setting	Has multifaceted utility	Utility
“The spectrum of ages covered is big. However, it doesn't specify if different responses should be expected according to the age”	Lack of guidance on age-appropriate expectations	Developmental specificity	Some further refinement needed	Utility
“Takes into account specific aspects of DoC in children”	It has been designed for use with children with DoC	Specificity	Rigor achieved through being evidence-based	Design

## 3 Results

Table 2 shows the respondents' roles in relation to children and young people with DoC and their levels of agreement with each statement. The respondent numbers are shown to enable referencing individual respondents in the thematic analysis.

**Table 2:** Respondents roles and levels of agreement with statements 1 and 2.

**Respondent ID**	**Role**	**Response to statement 1**	**Response to statement 2**
R1	Other healthcare professional	Agree	Strongly agree
R2	Other healthcare professional	Strongly agree	Agree
R3	Other healthcare professional	Agree	Agree
R4	Other healthcare professional	Agree	Agree
R5	Parent/carer	Strongly agree	Strongly agree
R6	Parent/carer	Strongly agree	Strongly agree
R7	Parent/carer	Strongly agree	Strongly agree
R8	Parent/carer	Strongly agree	Strongly agree
R9	Parent/carer	Strongly agree	Strongly agree
R10	Music therapist	Strongly agree	Strongly agree
R11	Music therapist	Strongly agree	Strongly agree
R12	Music therapist	Strongly agree	Strongly agree
R13	Music therapist	Strongly agree	Strongly agree
R14	Music therapist	Strongly agree	Strongly agree
R15	Music therapist	Strongly agree	Strongly agree
R16	Music therapist	Strongly agree	Strongly agree
R17	Other healthcare professional	Strongly agree	Strongly agree
R18	Music therapist	Strongly agree	Strongly agree
R19	Music therapist	Strongly agree	Strongly agree
R20	Music therapist	Strongly agree	Strongly agree

The total of 20 respondents was comprised of 10 music therapists, five parents with lived experience of caring for their child with a DoC, and five other healthcare professionals working in pediatric neurorehabilitation. Seventeen respondents were from the UK, two from Ireland and one from Spain. They each had in excess of 3 years of experience of caring for someone with a DoC. The other healthcare professionals included a speech and language therapist, an occupational therapist, a physiotherapist, a psychologist, and an educator. The table shows that all 20 respondents gave a response of “agree” or “strongly agree” to either statement. No other levels of agreement “strongly disagree,” “disagree,” and “undecided” were given by the respondents.

### 3.1 Overall outcome

#### 3.1.1 Statement 1: on initial review, the musicca appears to be an assessment of consciousness and awareness

**Figure 1:**
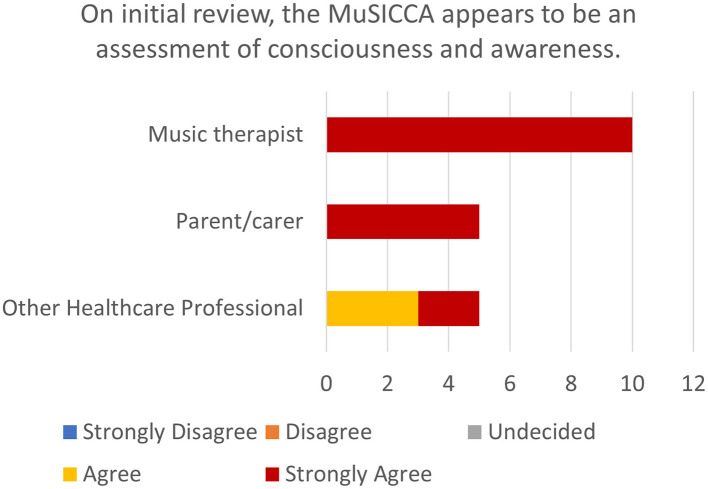
Stacked bar chart showing the number of ratings of levels of agreement with statement 1 by participants stratified by role.

The respondents showed 100% agreement that the MuSICCA appears to be an assessment of consciousness and awareness, with 85% strongly agreeing with the statement (see Figure 1).

#### 3.1.2 Statement 2: on initial review, the musicca appears to be suitable for use with children and young people

**Figure 2:**
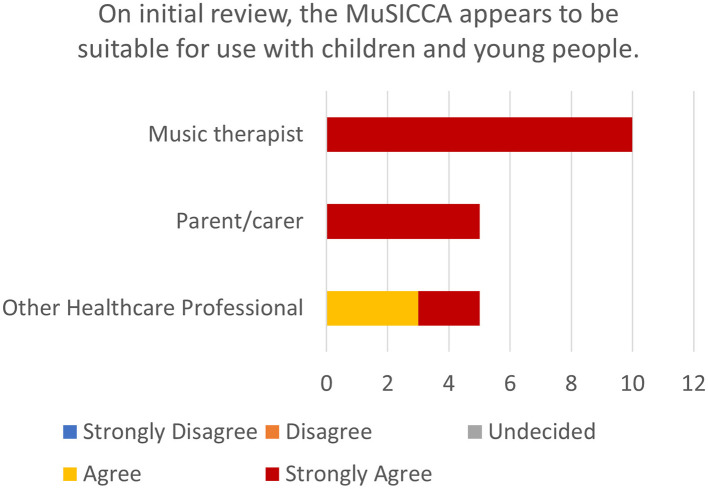
Stacked bar chart showing the number of ratings of levels of agreement with statement 2 by participants stratified by role.

The respondents showed 100% agreement that the MuSICCA appears to be suitable for use with children and young people, with 85% strongly agreeing with the statement (see Figure 2).

Inter-rater reliability for both statements was also assessed using Gwet's AC1. The Gwet's AC1 coefficient was 0.64, indicating substantial agreement among the 20 raters [95% CI (0.64, 0.64), *p* < 0.001]. These results confirm strong reliability among raters, supporting the consistency of ratings.

### 3.2 Thematic analysis of the responses

Table 3 shows the thematic map of the strengths and limitations of the MuSICCA.

**Table 3:** Thematic map of strengths and limitations of the MuSICCA.

**Domain**	**Superordinate theme**	**Theme**	**Sub-theme/ code**
Design	Rigor achieved through being evidence-based		
	Provides comprehensive assessment (design)		
Utility	Provides comprehensive assessment (utility)		
	Benefits and risks of caregiver involvement		
	Has multifaceted utility	Administration	
		Clinical	Acceptability
			Being a strengths-based approach
		Protocol	
		Informs treatment and goal setting	
	Required training reduces accessibility		
	Some further refinement needed	Guidance for implementation	
		Developmental specificity	

Two domains entitled Utility and Design emerged as organizing categories and contained all other thematic categories. Superordinate themes arose relating to each of these domains. In the domain of Design were “Rigor achieved through being evidence-based“ and ”Provides comprehensive assessment.” In the domain of Utility were “Benefits and risks of caregiver involvement,” “Has multifaceted utility,” “Required training reduces accessibility,” “Some further refinement needed.” The superordinate theme “Has multifaceted utility” Incorporated the themes of “Administration,” “Clinical,” “Protocol,” and “Informs treatment and goal setting.” The theme “Clinical” captured codes such as “Acceptability” and “Being a strengths-based approach.” The superordinate theme “Some further refinement needed” captured themes “Guidance for implementation” and “Developmental specificity.”

The above themes are described within the domains in which they are situated. Identifiers (R1, R2, R3, etc…) are used to indicate which respondents commented on the theme or code described.

#### 3.2.1 Design domain

##### 3.2.1.1 Superordinate theme: rigor achieved through being evidence-based

This superordinate theme captures the comments describing the design of the MuSICCA as based on evidence (research and clinical experience) and indicating the logical justification for aspects of the design. Respondents considered the MuSICCA to be based on research and a “good understanding” of children and young people with DoC (R1, R7, R16), considering specific aspects of the population (R16), seemingly “based on the experience of using measures” in pediatric DoC (R1). It was also felt that, as the MuSICCA is based on the MATADOC and was developed in consultation with a global network of music therapists experienced in pediatric DoC, it draws on relevant clinical practice (R19).

The respondents commented about the assessment protocol, suggesting that it seemed “strong, well-planned” (R6) and “flows naturally” (R10, R14), and that it “enabled identification of level of awareness” (R9).

In thinking about the MuSICCA's appropriateness for use in pediatric DoC, the respondents reported that it appeared “child-focused” (R6), using stimulation “that appeals to the children” (R7), “gives the opportunity for (responses) to take place” (R8), and involved a “reduced influence of complex language” (R12). They also mentioned that the MuSICCA's inclusion of parental voice as a stimulus enabled use of the “most salient auditory stimulus” (R16, R19) and including a child's family in assessment fostered closer working with “those who know the child best and can advise on best-known music” (R19).

##### 3.2.1.2 Superordinate theme: provides comprehensive assessment

Within the domain of “Design,” this superordinate theme encapsulates the comprehensiveness of sensory stimulation available to a music-based assessment tool. The respondents suggested that the MuSICCA protocol is thorough and covers all sensory modalities possible with musical stimuli (R1, R5, R9, R13). They highlighted the inclusion of the newly added “vibrotactile element to assess responsiveness” as a useful benefit (R11, R12, R15, R16, R20). “Gathering the pre-trauma story of the child's relationship with music” was mentioned as a strength (R6). The “methodology” of the protocol appeared comprehensive and “considered virtually all aspects of the situation for the individual” (R9).

#### 3.2.2 Utility domain

##### 3.2.2.1 Superordinate theme: provides comprehensive assessment

The comprehensiveness of the MuSICCA was captured in this superordinate theme from the perspective of clinical utility. Respondents stated that the inclusion of the vibrotactile element allowed for touch to be used strategically within the assessment. They reported that the MuSICCA appeared to provide a “thorough assessment of children and young people who might present with a DoC” (R1, R5, R9, R13) and it will “provide a thorough picture of how a young person is presenting” (R6, R13).

##### 3.2.2.2 Superordinate theme: benefits and risks of caregiver involvement

This superordinate theme represents both the strengths of caregiver involvement and some concerns expressed by the respondents. The possibility to include caregivers during the assessment was seen generally as a strength with one respondent suggesting that “children should have parents present during assessment” (R7). Respondents considered that parents might wish to be present and, therefore, the available guidance for including them in the assessment allows for caregivers to occupy a role (R3, R12) in their child's assessment through providing stimulation and child-specific knowledge that may “inform approaches within assessment sessions” (R20). However, one respondent acknowledged the need to address the duty of care to the caregivers when including them in assessments of their child (R6). Another noted that caregivers may not follow the guidance and, thus, may affect the assessment outcomes (R14).

##### 3.2.2.3 Superordinate theme: has multifaceted utility

The greatest number of comments were contained in this superordinate theme, captured by four themes (Administration, Protocol, Clinical, and Informs Treatment and Goal-Setting).

###### 3.2.2.3.1 Theme: administration

Comments within this theme refer to the rating form and documentation. These were considered to appear “user-friendly, clear, and simple” (R1, R13, R15) and able to be “completed in a timely manner” (R3, R4). The respondents highlighted the visual nature of the form and documentation and reported that this seemed helpful (R10, R16, R18). The electronic format of the rating form was seen as a strength and timesaving for clinicians (R11, R13, R15, R16, R18). The use of visual graphs was seen as a benefit for reporting on the assessment outcomes (R10, R11, R16). The respondents also liked the idea of the rating form feeding into a report to support the clinician when preparing reports (R11, R16).

###### 3.2.2.3.2 Theme: protocol

This theme captures the flexibility of the protocol for providing stimuli. Respondents suggested that the protocol allows for personally salient stimuli and familiar music that are “adaptable for individuals make this tool seem more likely to elicit responses” (R2) and increase the “chance for child engagement” (R3, R4). The respondents suggested that this adaptable use of music and sounds enables age-appropriate stimuli to be chosen that are based on the interests of the child or young person. For example, the respondents mentioned the use of nursery rhymes, children's familiar songs for young children, theme tunes and sounds from computer games for older children and young people as well as popular music (R4, R9, R16).

There were also comments on the protocol as a whole. Respondents suggested that it appeared to flow very easily (R10) and that its adaptable nature helped with assessment when attention might be limited (R14). The use of motor responses was seen as a strength (R16).

###### 3.2.2.3.3 Theme: clinical

Comments that centered on the clinical utility of the MuSICCA were encapsulated within this theme. These can be grouped into two sub-themes of Acceptability and Being a Strengths-based Approach. Relating to Acceptability, respondents felt that it had a “clear procedure based on the MATADOC” (R20) and that there are “a range of settings in which it can be used” (R6). However, the time burden of conducting four assessments was also mentioned (R6). The respondents viewed the MuSICCA as “strengths and abilities-based rather than a ‘test”' (R12, R16, R17). They highlighted this aspect of the measure and its relevance for treatment planning (R6, R10, R16). One respondent also noted that the MuSICCA protocol and refined rating system, utilizing the highest-rated response, reduced the impact of sensory impairments on the outcomes of the assessment (R12).

###### 3.2.2.3.4 Theme: informs treatment and goal-setting

This theme refers to the utility of the MuSICCA as an assessment tool that supports the team to develop goals and leads on to treatment. The respondents commented on specific characteristics of the MuSICCA, including its ability to record stimuli parameters (R10), its utility as a repeated measure for tracking progress and highlighting change (R10, R12, R17). One respondent mentioned the need to think carefully about reducing the number of sessions from 4 and how this might affect the diagnostic validity of the measure (R5). The MuSICCA was considered to complement other assessment tools through its music-based approach (R1) and, thus, support the multidisciplinary team through adding important information to build a comprehensive picture of the child or young person. The potential value of the MuSICCA to caregivers was also mentioned. One respondent stated that it appeared to be a “tool that will allow assessment and then go on to inform a treatment plan (this will/should offer reassurance to parents that assessment is meaningful and not an end state)” (R6).

##### 3.2.2.4 Superordinate theme: required training reduces accessibility

This superordinate theme relates to the accessibility of the MuSICCA as a music-based assessment that is conducted by a music therapist. One respondent highlighted the need for specific training and felt that “therapists using this tool would likely need quite a lot of individual teaching to be able to administer” (R2).

##### 3.2.2.5 Superordinate theme: some further refinement needed

Captured in this superordinate theme were the suggestions respondents provided around improvements and concerns. Concerns included comments about the reliance on the assessor's memory for recording the responses to stimuli (R16), and the observation that the protocol may not be able to be delivered exactly the same way each time due to the fact that live music is used in the protocol (R17). Many of the suggestions relate to the protocol instructions and the assessors' manual. Respondents felt that clearer guidance was needed on the presentation of visual stimuli in the protocol, specifically with reference to the positioning and timing of the movement of the visual stimuli (R3, R4). Clarification regarding the verbal command was also suggested for improvement (R10, R14, R20). The respondents commented that clarification in the guidelines is needed regarding the timing of the sessions, specifically referring to time of day and time after a rest period (R8, R9), to “ensure consistency of comparability” (R9). The respondents also suggested that the guidelines should contain clarification regarding expectations of responses at each age (R10, R12, R14, R20). One respondent highlighted the need to ensure comments by parents, carers, other staff are given significant attention to help build a comprehensive picture of the child or young person (R7).

## 4 Discussion

This study shows that the MuSICCA has sufficient face validity to be considered an assessment of consciousness and awareness that seems appropriate for use with children and young people. The 100% agreement between reviewers for both questions provides clear evidence for this statement. The answers from the respondents demonstrate the qualities of the MuSICCA in its design, and in its clinical utility as an assessment of awareness for children and youth with DoC.

The respondents highlighted the rigorous nature of the MuSICCA and that it is grounded in evidence. The adaptations for making it appropriate for use with children, the assessment protocol, and the use of salient stimuli (with potential inclusion of family) contribute to this rigor. These aspects of its design are congruent with recommendations for using music with children with DoC (Menén Sánchez et al., 2023; Bower et al., 2021) and enhance the MuSICCA's appropriateness and acceptability as an assessment tool.

Comprehensiveness was perceived by the respondents in this study as a particular strength in both its design and the breadth of its utility. This quality helps the assessor feel confident that the assessment is using all opportunities available to reveal signs of awareness in the patient. For the multidisciplinary team, this comprehensiveness enhances the relevance of the assessment information and can inform the approaches of other therapies (physiotherapy, speech and language therapy, and occupational therapy) for individual children. This can also support medical teams in their evaluation of the positive and negative effects of pharmacological treatments. The comprehensiveness and rigor of the behavioral assessment tool contribute to its diagnostic accuracy and to its ability to mitigate diagnostic confounds and to inform medicolegal evaluation (Zasler, 2024).

The use of music as a stimulus in the MuSICCA recruits widespread neural activation and cross-domain activity. These transient but powerful arousal-inducing (O'Kelly et al., 2013) and cognition-boosting effects (Schlaug et al., 2005; Castro et al., 2015) of music on the child's brain are the key to unlocking the child's potential to show awareness.

The MuSICCA addresses some of the recommendations set out in the practice guideline update for DoC (Giacino et al., 2018) including raising arousal prior to and during the assessment and using validated measures. This study is the first step in the process of producing a valid and reliable music-based assessment tool for children with DoC.

A particular strength of the MuSICCA is the protocolised involvement of family caregivers, if available, thereby maximizing the salience of the stimulation. This, to our knowledge, is the first assessment of DoC to actively involve caregivers, who are best placed to advise on stimuli that are the most familiar and salient, and who also hold detailed knowledge about the child's behaviors, personality, preferences, and experiences. Indeed, the auditory stimulus of a parent's or sibling's voice within the session may be the most familiar auditory stimulus for children and of itself increase arousal and responsiveness. Empowering parents by including them in the protocol delivery helps to enhance their own understanding of their importance as part of the team as their voice is the most salient stimulus helping to facilitate the child to show a response. Their knowledge about their child is critical to finding salient music and their involvement in the session enables greater accuracy in the interpretation of behaviors (Formisano et al., 2019). This might be understood to be somewhat contradictory to current common practice where caregivers' interpretations of behaviors in the DoC patient are given secondary, or even less, importance. However, this fits practice recommendations for the care of people who have a DoC (Leonardi et al., 2024, p. 231) and broader guidelines for family-centered pediatric neurorehabilitation. Involving the family in an assessment that is highly personalized may even help with their coping and the adjustment to the changes in their family's life. Including family members in this way also helps to build relationships with the healthcare providers. This is invaluable in their journey through the rehabilitation process and can facilitate discussion of multidisciplinary assessment findings with the family. Thus, the involvement of the family in the process may also support the multi-professional team in their work to support the child and the family.

The context of family-centered neurorehabilitation is important when considering face validity for this assessment tool since face validity may be defined as the degree to which the test appears relevant to the situation being considered. The broader context of this situation is one of optimizing care for a child or young person with highly complex needs and beyond merely providing diagnostic information. Involving the family in the protocol draws them immediately into the multidisciplinary team network of support and sits within the best practice guidelines that neurorehabilitation should be family-centered (Royal College of Physicians, 2020; Jenkin et al., 2023).

A recent study (Gosseries et al., 2023) investigating the needs, quality of life and emotional distress of caregivers of people with DoC showed that the needs for health information, professional support and involvement in care were the three highest rated by caregivers. This indicates the importance of these needs to caregivers of people with DoC. The MuSICCA addresses these needs by providing important information regarding responses to sensory stimuli, informing the multidisciplinary team to optimize their support for the patient and family, and proactively involving the caregivers in their child's rehabilitation.

The MuSICCA process helps to build a continuous thread of identity from before injury to the present, and to a future post-injury identity. This reflects existing music therapy brain injury rehabilitation protocols for adults that capture past and present musical identities to inform re-building of identity in treatment planning (Tamplin et al., 2016). Establishing the child's musical biography and the family's musical culture in the MuSICCA process can support psychological adjustment for the child and their family, aligning with models of family resilience during times of adversity (Perkins et al., 2022). The personalized nature of the MuSICCA assessment may help to mitigate the family's experience of losing the child's personhood (Løvstad et al., 2018) as part of a complex grief experience and the family's ambiguous loss. The process of participating in the MuSICCA protocol offers opportunities for the family to embody some psychological support (Leonardi et al., 2024) as it focuses on the child's personhood. The feedback of difficult news to family members regarding the diagnosis of DoC may also be mitigated since the parent has participated in the assessment and has developed a working relationship with the music therapist. Furthermore, the feedback from the MuSICCA highlights the child's individuality and identity. In clinical practice, feedback to the family often occurs with other team members who have completed complementary assessments and who have commented that the MuSICCA better captures the sense of the child and their personality.

Involvement in the MuSICCA protocol offers family members opportunities to witness moments of attunement during clinical improvisation. These moments occur when the music therapist responds to the child's breathing rate, movements, or vocalizations, and can lead to parental requests for family music therapy, guidance on how to interact musically with their child or discussions about parenting styles. This may further impact rehabilitation since nurturant parenting styles are associated with better outcomes for young people after an acquired brain injury (Root et al., 2016) and since finding meaningful ways of interacting can offer hope for families (Magee and Bowen, 2008; Menén Sánchez et al., 2023). The findings from this study indicate the potential utility of the MuSICCA not only for music therapy assessment, but for the entire multidisciplinary team, particularly in the absence of other developmentally relevant assessments for children and young people with DoC. The MuSICCA may offer a strengths-based assessment that supports the child or young person to show how responsive he/she is. As the protocol is personalized with a degree of adaptability to individual preference (Amari et al., 2017), salience can be maximized thus optimizing possibilities for responses. The information it generates informs the whole team's understanding of the child and how to optimize treatment, therapy, and care to meet the child's needs and set appropriate goals.

### 4.1 Limitations of the present study

The purpose of the present study of face validity was to gain a sense of whether there should be any further changes made to the MuSICCA prior to initiating more rigorous and detailed testing in the larger study that will follow. This larger, more rigorous study will evaluate the tool's construct validity, reliability (inter-rater and test-retest) and clinical utility. Despite this, it is important to acknowledge some of the limitations of the present study of face validity. The sample size for the face validity testing may be small when compared with evaluations of other types of validity that involve inferential statistical tests. This small sample size may have limited the credibility of the research findings. However, face validity has been poorly reported in previous research and there is a lack of reported methodologies with sufficient detail in both the DoC and music therapy literature in this area. Other studies have varied widely in their sample size. For example, Bower et al. (2023) recruited 10 healthcare professionals with experience working with DoC populations. In other fields of health research, a recent face validity study (Christian et al., 2025) has involved a sample size of <20 participants. A review of studies of face validity (Allen et al., 2023) showed a sample size range across studies of 7–76 participants. This present study's sample size falls within that range but is toward the lower end, and a larger sample size may have increased its rigor.

The composition of the sample may contain some bias, being made up of mainly music therapists, some non-music therapy healthcare professionals working in pediatric ABI settings, and some families of children with ABI. While this diverse group aided representativeness regarding the stakeholders of the MuSICCA, the larger proportion of music therapists may be considered to introduce bias into the sample. A justification for this is that it was necessary to limit the number of families that would be burdened by participation in the present study at a time when they might be experiencing significant psychological stress due to their child having a brain injury. Additionally, the key stakeholder group for the assessment of the tool are the clinicians who would be using it—the music therapists. They were considered most suitable to give specific feedback on the strengths and weaknesses of the MuSICCA and would be able to provide useful recommendations or suggestions for adaptations based on their unique knowledge of providing music interventions for children with DoC. Therefore, it was important to capture their opinions most of all. Thus, the sample was biased toward them. Despite the rationale given, the issue of bias would be addressed by having a larger sample in which each stakeholder group has equal numbers of participants.

The statistical analysis selected for this study was limited by the small sample size and the intention to focus on the opinions about the MuSICCA of the participants recruited rather than generalizing beyond the sample. The study of face validity is largely subjective as a construct and is based on the purpose of the individual tool and as such there is no one statistical index that measures face or content validity (Portney and Watkins, 2009). Therefore, face validity of the MuSICCA was considered to be sufficient if agreement between participants reached at least 75% agreement. In an editorial by Allen et al. (2023), the authors reviewed some studies that looked at face validity with the aim of providing some clarity regarding face validity and how it could be measured. Their review demonstrated that there is a lack of clarity around measuring face validity and, hence, a paucity of statistical recommendations for face validity in the literature. A larger sample size would have enabled the selection of more rigorous inferential statistics to increase the external validity of the results.

Further validation studies will be required in the future to test the psychometric properties of the MuSICCA, including predictive validity, and versions in other languages.

### 4.2 Conclusion

There is a paucity of assessment tools for pediatric DoC in the acute and sub-acute settings as a whole and this affects the entire clinical and care teams, not merely the music therapists. The consequences of misdiagnosis are very serious and discussions about the level of awareness have relevance for decision-making regarding withdrawal/continuation of hydration and nutrition, treatment/goal planning, designing, and providing optimal stimulation for rehabilitation, and access to rehabilitation in general. So, the whole team is affected by this need to accurately assess a child's level of consciousness following severe ABI. The MuSICCA—a music-based assessment tool for pediatric DoC—is uniquely designed to provide the optimal conditions to assess consciousness in children, and it possesses features that give the child the best possible chance of showing responses. The information from the assessment is directly relevant to the wider clinical team. The findings of this study of its face validity suggest that the MuSICCA possesses sufficient face validity in its current form to undergo more rigorous evaluation of its validity, reliability, and clinical utility with a larger sample size. This study, with perspectives from medical and therapeutic professionals as well as family caregivers, shows that the MuSICCA can be used to inform the whole team assessment of consciousness, goal setting and treatment planning for each child. Furthermore, it does so in a way that involves families who will potentially find the tool to be accessible and acceptable, particularly at a time of significant emotional stress and trauma for the family. The use of this music-based assessment tool can inform and support the whole multidisciplinary team to improve care and treatment for children with DoC.

## Data Availability

The raw data supporting the conclusions of this article will be made available by the authors, without undue reservation.
